# Strepsiptera, Phylogenomics and the Long Branch Attraction Problem

**DOI:** 10.1371/journal.pone.0107709

**Published:** 2014-10-01

**Authors:** Bastien Boussau, Zaak Walton, Juan A. Delgado, Francisco Collantes, Laura Beani, Isaac J. Stewart, Sydney A. Cameron, James B. Whitfield, J. Spencer Johnston, Peter W.H. Holland, Doris Bachtrog, Jeyaraney Kathirithamby, John P. Huelsenbeck

**Affiliations:** 1 Department of Integrative Biology, University of California, Berkeley, CA, United States of America; 2 Laboratoire de Biométrie et Biologie Evolutive, Université Lyon 1, Université de Lyon, Villeurbanne, France; 3 Departamento de Zoologia y Antropologia Fisica, Facultad de Biologia, Universidad de Murcia, Murcia, Spain; 4 Dipartimento di Biologia, Università di Firenze, Sesto Fiorentino, Firenze, Italia; 5 Fisher High School, Fisher, IL, United States of America; 6 Department of Entomology, University of Illinois, Urbana, IL, United States of America; 7 Department of Entomology, Texas A&M University, College Station, TX, United States of America; 8 Department of Zoology, University of Oxford, Oxford, England, United Kingdom; 9 Department of Biological Sciences, King Abdulaziz University, Jeddah, Saudi Arabia; Vanderbilt University, United States of America

## Abstract

Insect phylogeny has recently been the focus of renewed interest as advances in sequencing techniques make it possible to rapidly generate large amounts of genomic or transcriptomic data for a species of interest. However, large numbers of markers are not sufficient to guarantee accurate phylogenetic reconstruction, and the choice of the model of sequence evolution as well as adequate taxonomic sampling are as important for phylogenomic studies as they are for single-gene phylogenies. Recently, the sequence of the genome of a strepsipteran has been published and used to place Strepsiptera as sister group to Coleoptera. However, this conclusion relied on a data set that did not include representatives of Neuropterida or of coleopteran lineages formerly proposed to be related to Strepsiptera. Furthermore, it did not use models that are robust against the long branch attraction artifact. Here we have sequenced the transcriptomes of seven key species to complete a data set comprising 36 species to study the higher level phylogeny of insects, with a particular focus on Neuropteroidea (Coleoptera, Strepsiptera, Neuropterida), especially on coleopteran taxa considered as potential close relatives of Strepsiptera. Using models robust against the long branch attraction artifact we find a highly resolved phylogeny that confirms the position of Strepsiptera as a sister group to Coleoptera, rather than as an internal clade of Coleoptera, and sheds new light onto the phylogeny of Neuropteroidea.

## Introduction

Phylogenomic analysis — the application of dozens to many hundreds of alignments to phylogenetic problems — provides a better understanding of the phylogenetic relationships of species, by leveraging vast amounts of data. Indeed, many early simulation studies have suggested that a few thousand sites, a size typical of many phylogenetic analyses of a few genes, are inadequate to fully resolve a tree, especially if the problem is a difficult one [1], [2]. While the application of genomic data to phylogenetic problems is exciting, the field also poses profound problems for the analysis of these data. For example, historically, systematists sequenced the same gene, or ‘phylogenetic marker’, in multiple species and across laboratories. These genes were carefully selected for properties such as ease of alignment, an appropriate level of variation, and a low copy number in the genome [3]. With genomic data, on the other hand, the idea is to use a large number of the genes, even though their sequences may be difficult to align and analyse [4], and their history compounded with events of gene duplication, gene loss, and incomplete lineage sorting [5].

Computer simulation studies suggest that there may be another problem in phylogenomic analysis, statistical inconsistency. In cases where the alignments are very large *e.g.*, 100,000 sites [6] or even infinite in size [1], [7], the estimates of all the parameter values have very little (or no) associated uncertainty. Phylogenomic data sets have now reached such sizes, which means that if a phylogenetic method is inconsistent for a particular problem, the application of genome-scale data is likely to make the problem worse. As a consequence, careful attention must be paid to the modeling assumptions of the phylogenomic analysis.

The problem of inconsistent estimates of phylogenetic trees was first explored by [8] who described a combination of branch lengths on a four-species tree for which the parsimony method would converge to an incorrect estimate of phylogeny. The troublesome tree has two long branches separated by a small internal branch. The parsimony method strongly favors estimated trees in which the two long branches are incorrectly grouped together, leading to the adage that ‘long branches attract’ (in the following, we use “LBA” to stand for “Long Branch Attrraction” artifact). Later simulation studies showed that LBA is not limited to trees of 4 species, and may occur fairly frequently [9], [10]. Even though methods such as maximum likelihood, Bayesian inference, or distance methods, that correct for multiple substitutions on a branch, are less susceptible than parsimony to LBA [6], they can still become inconsistent when their model assumptions are misspecified and the problem is a difficult one.

Because the actual evolutionary history of any group cannot be directly observed, finding empirical examples of LBA is problematic. [11] investigated one possible example of LBA in the twisted-wing parasitoid order Strepsiptera. Historically, based on comparative morphology and a largely parasitic lifestyle, the order has usually been considered as related to Coleoptera, the order containing beetles, and possibly even inside Coleoptera, near other parasitic polyphagan families such as Ripiphoridae. In contrast, parsimony analyses of ribosomal DNA sequences resulted in a tree with Diptera and Strepsiptera as sister groups [12], [13]. The same analyses suggested an elevated rate of substitution in both groups, leading to the speculation that the long branches leading to the sampled Diptera and Strepsiptera were artifacts. Interestingly, maximum likelihood analyses of the same data placed Strepsiptera with Coleoptera. Moreover, a parametric bootstrap analysis of the data indicated that the branches were long enough to attract in a parsimony analysis. More recent studies that include more genes have consistently placed Strepsiptera with beetles [14], [15], although they usually did not include representatives of the coleopteran species proposed to be sister to Strepsiptera.

Here, we perform a phylogenomic analysis of insect data with several newly sequenced taxa with the goal of understanding if the LBA phenomenon associated with Strepsiptera remains a potential problem. We include new transcriptomes sampled from Coleoptera (4 transcriptomes, including the potentially related Ripiphoridae and Meloidae), Strepsiptera (2 transcriptomes), and Neuropterida (1 transcriptome). This improved taxonomic sampling allows us to ask several questions: are Strepsiptera within the Coleoptera, perhaps close to Ripiphoridae and Meloidae? If not, what is the position of Strepsiptera relative to Coleoptera and Neuroptera? The use of several methods and models of sequence evolution also enables us to investigate their performance on a difficult data set with a large amount of data, as large data sets can worsen LBA for susceptible methods.

### Strepsiptera Biology and Phylogeny

**Figure 1:**
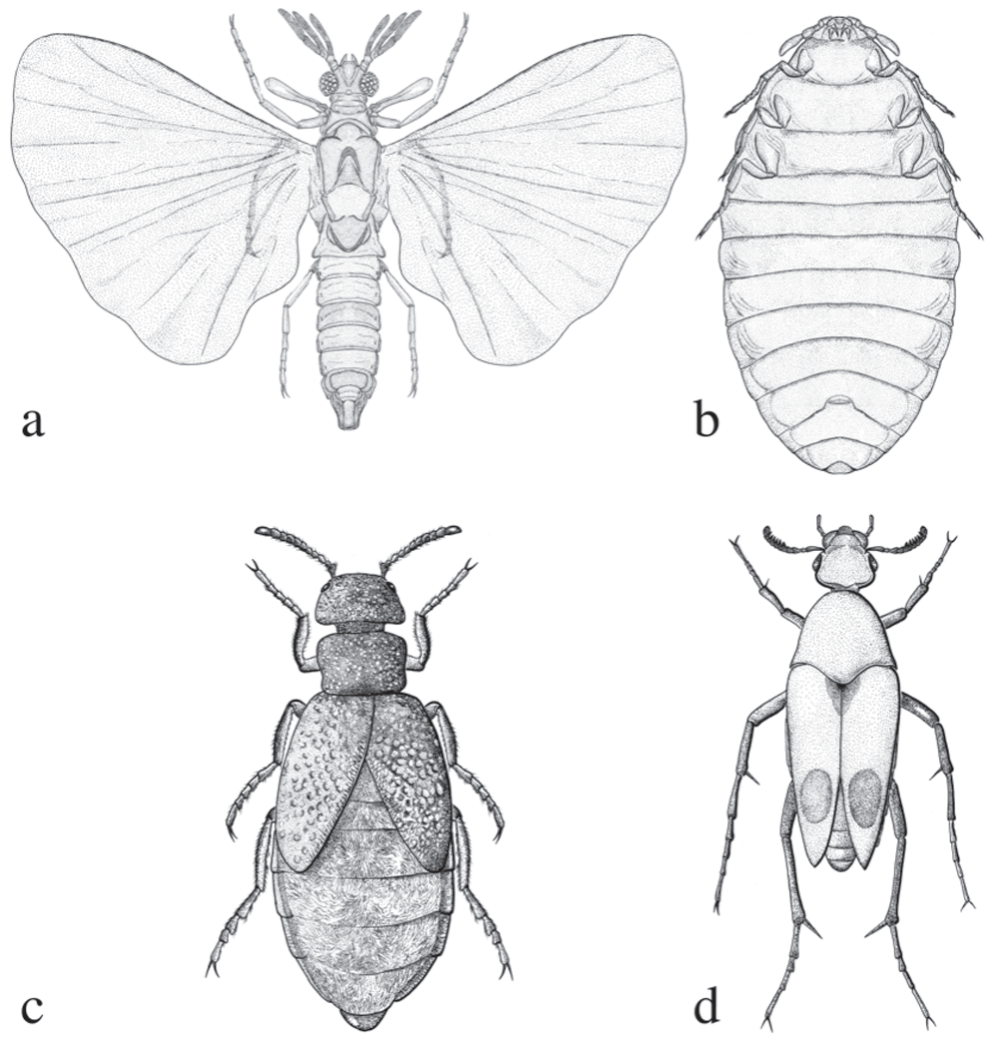
Representations of a male *Eoxenos laboulbenei* De Peyerimhoff (Strepsiptera), dorsal view (a), neotenic female *Eoxenos laboulbenei* De Peyerimhoff (Strepsiptera), ventral view (b), *Meloe brevicolis* (Panzer) (Meloidae, Coleoptera), dorsal view (c), *Macrosiagon tricuspidatum* (Lepechin) (Ripiphoridae, Coleoptera), dorsal view (d). Drawings by Juan A. Delgado.

Strepsiptera have fascinated biologists from the time they were first described by [16]. [17], who studied *Xenos vesparum* (Stylopidia), sums up his own observations: “*Quoi qu'il en soit, cet insecte est un des plus singuliers et des plus intéressants que puisse offrir la nature.*” (“This insect is one of the strangest and most interesting that nature can offer”). Strepsiptera have been divided in two major groups, Mengenillidia and Stylopidia. Both are obligate entomophagous parasitoids during most of the larval stages and exhibit a variety of unusual phenotypic features [18]–[25]. Stylopidia exhibit extreme sexual dimorphism: the males remain endoparasitic in their hosts to pupate, emerging as free-living adults, but the females remain endoparasitic as neotenic adults and have no distinct head, thorax or body appendages [18]–[20], [22], [25]. In contrast, in Mengenillidia both sexes leave their hosts before pupation and are free-living as adults, and the females possess all the body appendages typical of an insect, except wings (Fig. 1a,b). Mengenillidia and Stylopidia also differ in their reproductive practices: in Mengenillidia the free-living females are fertilized by traumatic insemination, whereas females of Stylopidia are inseminated through the brood canal opening [18]–[23], [25], [26].

Strepsiptera also display distinctive genetic characteristics. They have extremely small genomes [27], very unusual insertions in their 18S ribosomal DNA sequences [28], and they have undergone high rates of sequence evolution [24]. These insertions and high substitution rates have contributed to the difficulty in placing Strepsiptera in the insect phylogeny, a problem sometimes called the “Strepsiptera Problem” [29].

On the basis of morphological and genetic characters, Strepsiptera have been said to be: (i) akin to Hymenoptera [16], (ii) akin to Diptera [12], [30]–[33], (iii) a sister group to Coleoptera [14], [15], [18], [20], [23], [25], [34]–[36], (iv) placed within the Coleoptera [35], [37]–[39], and in particular close to meloid beetles (Fig. 1c) or ripiphorid beetles [40] (Fig. 1d), and (v) accorded an ambiguous placement as *Neoptera incertae sedis* [41]. In the past 15 years alone, molecular studies have placed Strepsiptera in 4 different positions [11], [14], [15], [32], [34]–[36].

Recently [36] sequenced the nuclear genome of a species of Strepsiptera and compared it to genomic or transcriptomic data from 12 other insect species, including two Coleoptera (beetles). Commonly-used methods of tree reconstruction using either amino-acid or recoded DNA data yielded a phylogeny in which Strepsiptera are the sister group to Coleoptera. The large quantity of sequence information contained in their data set as well as the resulting high support found on all nodes of their phylogeny led the authors to conclude that the Strepsiptera enigma has been resolved. However this phylogeny did not include a member of Neuropterida, usually sister group to Coleoptera, nor did it include representatives of groups within Coleoptera previously hypothesized as close relatives of Strepsiptera. In addition, a phylogeny obtained with a large number of sites but a small number of taxa may fall prey to known artifacts of phylogenetic reconstruction, in particular to LBA. Therefore it is not quite clear whether Strepsiptera form a group within Coleoptera, are sister group to Coleoptera, to Coleoptera+Neuropterida, or to Neuropterida, a result notably obtained based on seven nuclear protein-coding genes by [35]. As a result, in their review [42] they consider the monophyly of Coleoptera and Strepsiptera as “tenuously supported”. Further, a recent study comparing transcriptomic and morphological data concluded that the “monophyly of Coleopterida (Coleoptera and Strepsiptera) remains ambiguous in the analyses of the transcriptome data, but appears likely based on the morphological data.” [43].

Besides Strepsiptera, many other groups of parasitic or parasitoid organisms have been the topic of similar phylogenetic controversies. In fact, such organisms tend to show high rates of morphological and molecular evolution, complicating phylogenetic reconstruction. In recent years however, several controversies surrounding fast-evolving species have been resolved. Examples include the placement of Urochordates as sister group to Vertebrates [44], the placement of microsporidia as fungi [45], the placement of nematodes as Ecdysozoa [46], [47]. In all cases, the use of better models of sequence evolution and adequate taxonomic sampling corrected the LBA, and changed the position of rapidly evolving taxa from outside existing clades in the phylogeny to inside them.

In the case of Strepsiptera, their high rate of sequence evolution [11], [24] makes them good candidates for falling prey to LBA. As a consequence, it is important that a large number of species and robust models of sequence evolution be used to resolve their phylogenetic position. We gathered and generated large amounts of sequence data with deep taxonomic sampling, and we used models of sequence evolution that have been shown to be robust against LBA. We used the recently sequenced transcriptomes of eight beetle species [48], the recently sequenced genome of a mengenillid (Strepsiptera) [36] and genomic data for other insects downloaded from publicly available databases. In addition, we sequenced the transcriptomes of two species of Strepsiptera, a mengenillid *Eoxenos laboulbenei* De Peyerimhoff, and a xenid, *Xenos vesparum* (Rossi) representing the deepest divergence in this group, four species that represent the major groups of Coleoptera, and one lacewing, belonging to Neuropterida, often found to be sister group to Coleoptera in insect phylogenies [42]. We translated our sequence data into amino acids, which have been found to be more robust against reconstruction artifacts [49]. We used both maximum parsimony and model-based approaches to address both the influence of the data set and the influence of the model of sequence evolution on the inferred phylogeny. Notably we used site-heterogeneous models of sequence evolution [50] that account for the variety of biochemical contexts surrounding sites in proteins and are robust against LBA.

## Results and Discussion

### Parsimony analyses

**Figure 2:**
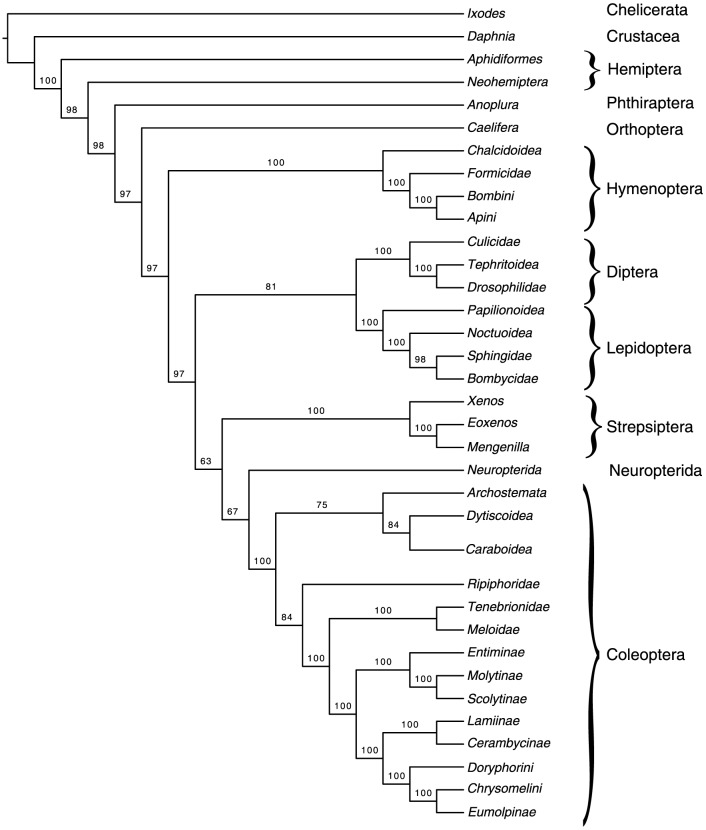
Phylogenetic tree reconstructed using Maximum Parsimony.

Early molecular analyses of ribosomal RNAs supported a close proximity between Diptera and Strepsiptera [12]. This result was found due to the use of parsimony where the assumption of an absence of multiple substitutions is violated by the data, and to be consistent with LBA [11]. We investigated whether using the same method on a much larger amino-acid data set could recover similar results. We used PAUP* with default parameters to run a parsimony analysis on the entire data set. A single most parsimonious tree was recovered (Fig. 2), 362,884 steps long, placing Strepsiptera outside of Neuropteroidea (Coleoptera + Neuropterida) [42]. Bootstrap analysis (1,000 bootstrap replicates) resulted in 1026 trees. Of these, Strepsiptera were found 197 times next to Diptera, and 343 times next to a group containing Diptera and Lepidoptera. This suggests that the signal in early studies recovering Strepsiptera next to Diptera based on maximum parsimony analysis of ribosomal RNA molecules is also present in a weaker form in our large alignment of protein-coding genes. Among the bootstrap replicates, Strepsiptera were also found 335 times sister to Neuropterida, and 316 times next to Neuropteroidea, but were never found next to Coleoptera or inside Coleoptera. Constrained analyses with either Strepsiptera inside polyphagan beetles or Strepsiptera next to beetles resulted in longer trees with 363,182, and 362,965 steps, respectively. These maximum parsimony analyses of our phylogenomic data set therefore do not agree with the series of recent results that place Strepsiptera with Coleoptera.

### Model-based analyses

Accurate models of sequence evolution are key to a reliable phylogenetic reconstruction. Model choice is usually accomplished through a comparison of candidate models, and the model with the best relative fit is chosen. Such a choice can be accomplished using Bayes Factors, likelihood ratio tests, or Akaike or Bayesian Information Criteria *e.g.* [51]–[53]. However these approaches are highly dependent upon the set of candidate models considered, and do not provide a measure of the absolute fit of the model to the data. Alternatively, posterior predictive tests provide such an absolute measure. They are based on the idea that a model that fits the data should be able to generate the data, and they work by comparing summary statistics computed on the true alignment to summary statistics computed on alignments simulated under the model [54]–[56]. The choice of the summary statistic defines the characteristics of the data that the practitioner deems most important. In our case, as we are concerned that LBA may be affecting the position of Strepsiptera in the insect phylogeny, we use as summary statistic the observed diversity (the number of different amino-acids per site of the alignment) detected by the model in the data.

We used two types of models on our data set: models that are homogeneous among sites, which have previously been used to study the insect phylogeny [11], [14], [15], [32], [34]–[36], and models that are heterogeneous among sites, in which sites are assumed to come from a mixture of models. The use of homogeneous models enables us to address the impact of our data set on phylogenetic inference, and the use of heterogeneous models to address the impact of models that have been shown to be efficient at reducing LBA [56]. We fitted both GTR+Γ [57] and LG08+Γ [58] homogeneous models and we also fitted two site-heterogeneous models: CAT+Γ and CATGTR+Γ [50]. We used PhyloBayes to run all four models and estimate their fit (Lartillot et al., 2009). Among the four models, LG08+Γ and CATGTR+Γ had convergence issues. Despite having run the CATGTR+Γ chains for more than 2000 iterations, the maximum difference in bipartition split difference was about 0.2, and one chain obtained with the LG08+Γ model seemed to be trapped in a local maximum. For the CATGTR+Γ model, we report posterior predictive tests for each chain. We will not discuss the LG08+Γ model further. Interestingly all models are rejected as they are unable to reproduce the site-wise diversity observed in the data. The site-homogeneous models overestimate the site-wise diversity with a value of 3.90 compared to 3.33 in the real data (*p*-value  = 0). With CATGTR+Γ, the overestimation is less pronounced, but still significant at least for one of the two chains (values of the statistic 3.75 and 3.61, p-values of 0 and 0.07 respectively). Finally, CAT+Γ *underestimates* the site-wise diversity, with a value of 2.70 (*p*−value  = 1.00). These posterior predictive tests indicate that site-homogeneous models and, to a lesser extent the CATGTR+Γ model, may fall prey to LBA, but the CAT+Γ model may overcorrect against LBA. It is not clear what may be the impact on phylogenetic reconstruction of overcorrecting against LBA. However, if both models that undercorrect and models that overcorrect against LBA provide the same tree topology, one may be hopeful that LBA is not strongly affecting the topology.

**Figure 3:**
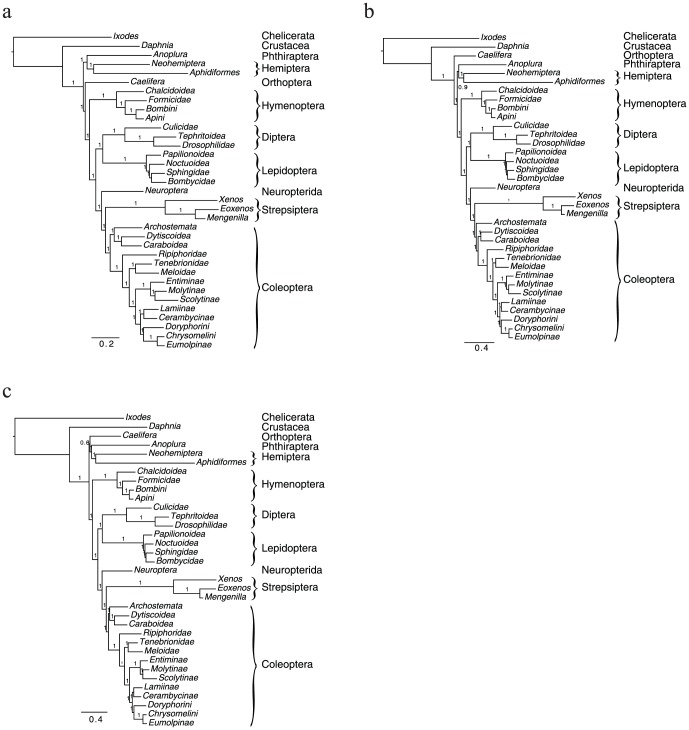
Phylogenetic trees reconstructed using GTR+Γ (a), CAT+Γ (b) or CATGTR+Γ (c).

GTR+Γ, CAT+Γ and CATGTR+Γ support nearly identical phylogenies for our 36 species, in excellent agreement with the current consensus insect phylogeny [42], and with high support (Fig. 3). However, the three models disagree in two areas of the tree. First, they disagree on the relative arrangement of Orthoptera and Paraneoptera (Phthiraptera and Hemiptera). GTR+Γ places Orthoptera closer to holometabolous insects, with high confidence, whereas CAT+Γ places it further from holometabolous insects, also with high confidence. CATGTR+Γ places Orthoptera as sister group to Paraneoptera, but with very low confidence, perhaps because the chains have not quite converged under this model. This disagreement, even among the two site-heterogeneous models, confirms that this part of the tree of Arthropods is still unresolved [42]. These three models also disagree on the placement of the basal clades of beetles, Archostemata and Adephaga (the latter represented in our tree by Dytiscoidea and Caraboidea). GTR+Γ and CATGTR+Γ place Archostemata and Adephaga as sister groups, whereas Archostemata diverge first in the CAT+Γ tree, in agreement with the analysis by [59] in a study of one to three genes for nearly 1900 species. Both of these unresolved areas of the insect tree arise in clades that are vastly under sampled. While our data comprising hundreds of genes covers a broader phylogenetic diversity of Coleoptera compared to recent studies, we are still far from sampling much of the beetle diversity. Our sampling of Hemiptera, Phthiraptera and Orthoptera is also limited. Analyses focused on these specific phylogenetic problems, with targeted taxonomic sampling, will likely provide improved resolution.

Among the different methods, branch lengths vary markedly, with homogeneous models, for example GTR+Γ yielding branch lengths on average 1.7 or 1.3 times smaller than CATGTR+Γ and CAT+Γ, respectively. For the longest branches, for instance, the branch leading to Strepsiptera, the fold differences are larger, as this branch is 2.39 and 2.12 times smaller in the GTR+Γ tree than in the CATGTR+Γ and CAT+Γ trees, respectively. These statistics confirm that the site-homogeneous models may be more susceptible to mistaking homoplasies for synapomorphies. However, it is unclear which model among the three we tested most accurately estimates the true expected numbers of substitutions in our data set. Such uncertainty could lead to problems for analyses aimed at dating divergence events, especially in the vicinity of long branches, and may deserve further investigation.

All the models we used make several unrealistic assumptions regarding the process of sequence evolution. Notably, they assume that the process has been homogeneous across branches, an assumption rejected by a posterior predictive test where compositional heterogeneity among sequences is measured (*p*−value  = 0 for all models). Compositional heterogeneity across sequences can mislead phylogenetic reconstruction. Currently, no model able to deal with both heterogeneity across branches and heterogeneity across sites for data sets this size has been published. However, an alternative approach that has been shown to be successful against both LBA and compositional heterogeneity is recoding of the data [49], [60]–[62], so that amino acids with similar biochemical properties are grouped together, and only substitutions between groups are taken into account for phylogenetic reconstruction. We used three different recoding schemes, in six, four and two categories. All three recover Strepsiptera sister group to Coleoptera with high support, and confirm the results obtained with the other models (data not shown). These results show that compositional heterogeneities are unlikely to be causing the placement of Strepsiptera outside Coleoptera.

All model-based analyses agree on the result that Strepsiptera are sister group to Coleoptera, and further that Neuropterida is sister group to those two. However, parsimony analyses place Strepsiptera sister to a group containing Neuropterida and Coleoptera. Given the high rates of sequence evolution observed in Strepsiptera, it is possible that this latter result is a manifestation of LBA, as was the early placement of Strepsiptera next to Diptera. However, beyond LBA, several properties of the data may mislead phylogenetic reconstruction under the parsimony criterion: for instance compositional heterogeneities among sequences or rate heterogeneities among sites could be problematic. Overall, although it is difficult to understand what factors led parsimony to such an unconventional result, it is difficult to put the blame on a particular, specific artifact [63]. In any case, this result serves as a reminder that increasing the quantity of data is not a cure for model misspecification. Model-based methods that account for site heterogeneities therefore confirm and add precision to those from [36], with better taxonomic sampling from both Coleoptera (including previously hypothesized sister groups to Strepsiptera) and Strepsiptera, and with a species from Neuropterida. Features shared by Strepsiptera and Coleoptera such as enlarged hindwings and immobile mandibles of the pupa are most likely shared by common ancestry. Other characteristics found in Strepsiptera and only some families of polyphagan Coleoptera (including Riphophoridae), such as the active host-seeking 1st instar larvae, the many branched antennae, partially reduced mouthparts and heteromorphosis, are likely due to evolutionary convergence. Our finding of a sister group relationship between Neuropterida and Coleoptera+Strepsiptera contradicts [35] 's results from 7 nuclear protein-coding genes, which was found to be unlikely based on morphological grounds [36]. However it agrees with another of their analyses including the same seven genes plus two nuclear ribosomal RNAs. Given the very high support found in our analyses for this relationship, and the relatively small branch length leading to Neuropterida, it seems likely that this result will hold, even when more sequences are included. In addition, our more specific confirmation that Strepsiptera do not have closest relatives within Coleoptera, but are instead sister to it, is significant in that it confirms that Strepsiptera remains a valid distinct order of insects. Although finding the phylogenetic position of lineages with high rates of sequence evolution and highly derived lifestyles and morphologies is often challenging, all the recent genomic evidence and analyses point with very high support to Strepsiptera as sister group to the Coleoptera, and Neuropterida sister group to those two.

## Materials and Methods

### Collection of insect specimens

Specimens were collected as shown in Table 1. We also downloaded from public databases genomic and transcriptomic data for 19 other species of insects, and additionally used data from recently published works [36], [48].

**Table 1:** Collection sites for specimens used in the present study.

Species	Collection
*Priacma serrata* (Le Conte)	USA, Blodgett forest,
Coleoptera (Archostemata)	38°54′23.47″N 120°39′33.63″W,
(most likely ♂as they were attracted to bleach)	14.06.2011(hand collected)
	(J. Huelsenbeck)
*Chrysoperla rufilabris* (Bumeister)	USA, Berkeley, 18.05.2011
(Neuropterida: Chrysopidae) (larva)	(eggs reared on *Drosophila melanogaster*)
	(B. Boussau)
*Thermonectes intermedius* (Crotch)	USA, near Sacramento
(Coleoptera: Dytiscidae) (adult)	39°16′44.22″N 122° 7′0.08″W
	04.09.2010 (Doug Post, State of California, Department of Fish and Game, Water Pollution Control Laboratory, 2005 Nimbus Road, Rancho Cordova, CA 95670).
*Xenos vesparum* (Rossi)	SPAIN, Forest of *Pinus halepensis*, Sierra Espua Natural Park, near Huert Espua, Murcia.
(Strepsiptera: Xenidae) (neotenic ♀adult)	09.07.2010
	37° 51′27.20″ N, 1°31′10.46″W
	(hand collected) (J. Kathirithamby, J. Delgado, F. Collantes)
*Eoxenos laboulbenei* De Peyerimhoff	SPAIN, Land Farm, apricot, orange and lemon orchards and farrow land,
(Strepsiptera: Mengenillidae) (♂adult)	on road from Mula to Pliego, Murcia,
	38°00′25.27″N 2°28′2.46″W,
	06–08.09.2011 (light trap)
	(J. Kathirithamby, J. Delgado, F. Collantes)
*Meloe brevicollis* (Panzer)	SPAIN, Santuario de Cristo, near Moratalla, Murcia
(Coleoptera: Meloidae) (adult)	38°10′46.16″N 2°04′41.56″W,
	3.10.2010, (hand collected)
	(J. Delgado)
*Macrosiagon tricuspidatum* (Lepochin)	USA, IL, Saline Co., State Fishand Wildlife Conservation Area,
(Coleoptera: Rhipiphoridae)	Shawnee national Forest, Glen O. Jones Lake
(adult)	on *Erigeron philadelphicus*,
	37°41′16.01″ N 88°23′29.10″W,
	8.06.2010 (I. Stewart)

### RNA extraction

Total RNA was purified with commonly-used Trizol/Chloroform purification protocols. Library preparation was done as recommended by Illumina, with custom-order primers from IDT (based on Illumina's description of their primer and adapter sequences). The library for *Eoxenos laboulbenei* (Mengenillidia) was prepared at the Beijing Genome Institute from total RNA extracted as for other samples, all other libraries were prepared at UC Berkeley.

### Transcriptome sequencing

Sequencing of paired-end 100 bp fragments was done on Illumina Hiseq sequencers.

### Transcript assembly

We used Trinity [64] to assemble reads into putative transcripts for the six de-novo sequenced transcriptomes. These putative transcripts can be downloaded from http://dx.doi.org/10.6084/m9.figshare.1040412 or from ftp://pbil.univ-lyon1.fr/pub/boussau/StrepsipteraPaperData/.

### Clustering into families of homologous genes

Transcript sequences were translated into protein sequences with the script “transcripts_to_best_scoring_ORFs.pl” from the Trinity package [64]. We used blastp all-against-all to compute similarities among all proteins in our data set and silix [65] to cluster sequences in groups of homologous sequences. We changed the minimum percent of overlap to 30% to accept partial transcripts produced by Trinity in families.

### Definition of families of orthologous genes

First, we selected families with more than 20 and less than 100 sequences. For each family, we generated an alignment using MAFFT [66] with the following options: “–maxiterate 1000 –localpair –anysymbol –thread 1”. Then a Fasttree [67] phylogenetic tree was generated for each alignment using default options. Then we used an in-house program to prune the alignments from species-specific duplicates, merging the sequences when they were not entirely overlapping (program available upon request). This resulted in 668 gene families, or 192,807 sites in total. We added to this data set another data set based on families in which one species is represented by two non-monophyletic sequences. For these families we removed the shortest duplicate. This second data set resulted in 549 gene families and 272,093 sites.

### Removal of putative contaminants

Contaminant sequences may have been introduced in our data sets during sequencing, but could also correspond to paralogous (descending from a duplication event) or xenologous (coming from a gene transfer event) sequences that have been included in our putative families of orthologous sequences. We used Phylo-MCOA [68] with patristic distances and default parameters to filter out contaminant sequences from the 1217 gene families. No species was found to be a “complete” outlier, but 7 gene families were found to be “cell-by-cell” outliers and were therefore removed. In addition, 1607 genes were removed from the gene families.

### Concatenation

The alignments were first concatenated into two supermatrices corresponding to the two data sets. Then Fasttree [67] phylogenetic trees were generated from these two supermatrices. Based on these trees, we found no evidence for incompatibility between the two alignments and decided to concatenate all alignments together into a single supermatrix of 446,428 positions. We applied Gblocks [69] with the following parameters “minimum number of sequences for a conserved or flank position: 14; maximum number of contiguous nonconserved positions: 8; minimum length of a block: 10; allowed gap positions: all” on the supermatrix, which resulted in an alignment with 92,836 amino-acid positions. The median amount of missing data was 21.7% (1st quantile 14.2%, 3rd quantile 54.6%). This supermatrix can be downloaded from http://dx.doi.org/10.6084/m9.figshare.1040412 or from. ftp://pbil.univ-lyon1.fr/pub/boussau/StrepsipteraPaperData/.

### Phylogenetic analyses

Phylogenetic analyses and posterior predictive tests were run with PhyloBayes [70]. Convergence was decided using bpcomp from the PhyloBayes package by comparing two chains per model when the maximum difference in node posterior probabilities between the two chains was below 0.1.
